# Overexpression of angiotensin-converting enzyme in myelomonocytic cells enhances the immune response

**DOI:** 10.12688/f1000research.7508.1

**Published:** 2016-03-23

**Authors:** Kenneth E. Bernstein, Zakir Khan, Jorge F. Giani, Tuantuan Zhao, Masahiro Eriguchi, Ellen A. Bernstein, Romer A. Gonzalez-Villalobos, Xiao Z. Shen

**Affiliations:** 1 Department of Biomedical Sciences and the Department of Pathology, Cedars-Sinai Medical Center, Los Angeles, CA, USA

**Keywords:** Angiotensin-converting enzyme, ACE, angiotensin, immune response, renin-angiotensin system

## Abstract

Angiotensin-converting enzyme (ACE) converts angiotensin I to the vasoconstrictor angiotensin II and thereby plays an important role in blood pressure control. However, ACE is relatively non-specific in its substrate specificity and cleaves many other peptides. Recent analysis of mice overexpressing ACE in monocytes, macrophages, and other myelomonocytic cells shows that these animals have a marked increase in resistance to experimental melanoma and to infection by *Listeria monocytogenes *or methicillin-resistant *Staphylococcus aureus *(MRSA). Several other measures of immune responsiveness, including antibody production, are enhanced in these animals. These studies complement a variety of studies indicating an important role of ACE in the immune response.

## Introduction

The renin-angiotensin system (RAS) is composed of the substrate angiotensinogen, a protein produced by the liver, which is sequentially degraded by the enzymes renin and angiotensin-converting enzyme (ACE) into the vasoconstrictor angiotensin II. The workings of this system and its role as a central regulator of blood pressure control have been the subject of thousands of scientific publications ^1^. Although there are voluminous biochemical data on each of the components of the RAS, an interesting question is the origins of this system. Such a question recognizes that blood pressure is a function of higher organisms, whereas the origins of the proteins composing the RAS go back to a time when simpler organisms had neither blood nor blood pressure. Angiotensinogen is a liver acute-phase protein; it is an α2 globulin related to haptoglobin and α2-microglobulin. Renin, made in the kidney, is an aspartyl protease related to pepsin, whereas ACE, made by endothelium and many other tissues, is a zinc-dependent peptidase. Whereas angiotensinogen and renin have evolved to the point where they participate only in blood pressure regulation, ACE plays additional roles and has been implicated in normal male reproduction, several aspects of hematopoiesis, the degradation of β-amyloid peptides, and several other physiologic and pathologic processes ^2– 7^.

One of the most interesting areas of ACE biology is its role in the immune response. Early evidence for this came from the analysis of ACE expression by the macrophages and giant cells found in granuloma. The formation of a granuloma is the immune system’s response to organisms or inorganic materials that are difficult to phagocytize ^8^. The classic granuloma is associated with tuberculosis, but granuloma formation is also seen in sarcoid, leprosy, and several other diseases. It has been known for many years that the epithelial macrophages and giant cells that are a prominent feature of granulomas express ACE ^9^. Thus, there is an association between the immune activation of the monocytic-derived cells in a granuloma and ACE expression. What remained unknown was whether ACE expression was also a contributor to this process.

## ACE 10/10 mice

**Figure 1.**
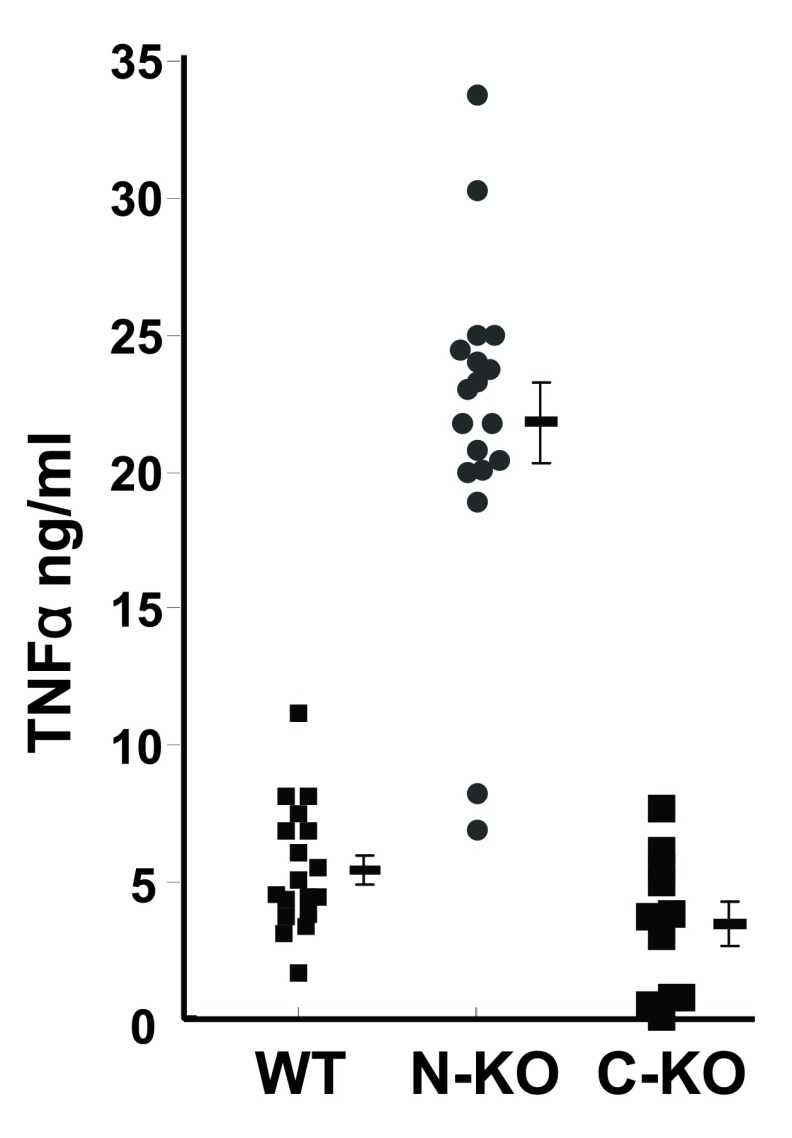
Tumor necrosis factor-alpha (TNFα) expression in macrophages lacking the angiotensin-converting enzyme N- or C-catalytic domain. Thioglycolate-elicited peritoneal macrophages were collected and purified by adhesion. After an overnight incubation with 1 μg/mL lipopolysaccharide, the concentration of TNFα was determined by enzyme-linked immunosorbent assay. The figure shows data for individual mice and the group mean ± standard error of the mean. C-KO, **macrophages lacking the angiotensin-converting enzyme C-catalytic domain**; N-KO, **macrophages lacking the angiotensin-converting enzyme N-catalytic domain**.

Our experience with ACE and the immune response first came from the study of mice with genetic mutations in the ACE gene. The wild-type (WT) ACE protein contains two independent catalytic domains that resulted from an ancient gene duplication, estimated to have occurred over 300 million years ago ^1^. Each catalytic domain independently binds zinc, which is required for catalytic activity. To understand the function of each catalytic domain, mice were made in which point mutations were introduced into the mouse genome that specifically inactivated either the ACE N- or C-domain zinc binding site ^10, 11^. Thus, the mutations specifically inactivated the N- or C-domain catalytic sites. These mice are called ACE N-KO and ACE C-KO, respectively. In these animals, the non-targeted domain remained fully catalytic and the tissue expression levels of the mutated ACE proteins were identical to those of WT mice. When peritoneal macrophages from these animals were isolated and exposed *in vitro* overnight to lipopolysaccharide (LPS), the macrophages were activated and secreted tumor necrosis factor alpha (TNFα) into the media ( Figure 1) ^12^. While mice lacking ACE C-domain catalytic activity produced roughly equivalent levels of TNFα as WT mice, macrophages from mice lacking N-domain activity (ACE N-KO) produced approximately fourfold higher levels. Thus, ACE activity can have profound effects on the ability of macrophages to produce the important pro-inflammatory cytokine TNFα.

A second model giving insight into the role of ACE in the immune function is a genetically engineered mouse line termed ACE 10/10. Here, gene targeting was used to insert a neomycin resistance cassette and strong transcriptional stop signal 3′ to the ACE promoter ^13^. This acts as a barrier to endogenous ACE promoter activity. 3′ to the neomycin cassette, we inserted a *c-fms* promoter cassette that now controls tissue expression of the ACE gene. c-fms encodes the receptor for macrophage colony-stimulating factor and is normally expressed at high levels by myelomonocytic lineage cells ^14, 15^. Mice that are heterozygous or homozygous for the ACE 10/10 allele overexpress active ACE in monocytic cells such as monocytes and macrophages. Other myelomonocytic cells, such as neutrophils and dendritic cells, also overexpress ACE but at only 4% and 17% of macrophage levels, respectively. ACE expression by T or B cells is very low, similar to WT mice. Homozygous ACE 10/10 mice lack ACE expression by endothelial cells and renal epithelial cells. Despite this, ACE 10/10 mice have normal body weights, serum ACE levels, renal function, and blood pressure. They live normal life spans and have no evidence of auto-immune disease.

**Figure 2.**
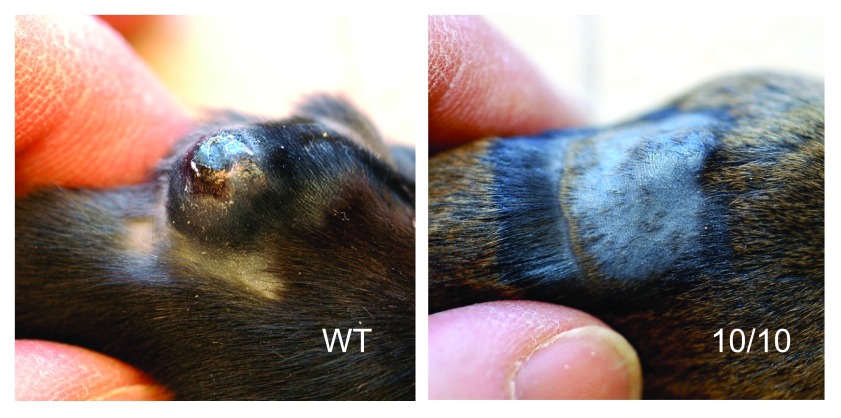
Growth of the B16 melanoma in angiotensin-converting enzyme (ACE) 10/10 mice. Both ACE 10/10 and wild-type (WT) mice were injected intradermally with one million B16-F10 melanoma cells. After 14 days, there was a very significant difference in tumor growth, with WT mice having much larger tumors than ACE 10/10 mice. These photos show typical results. The fur of the ACE 10/10 was shaved to demonstrate the reduction in tumor size in these animals.

The first inkling that ACE 10/10 mice were unusual came from challenging the mice with B16-F10 (H-2 ^b^) melanoma tumor cells ^13^. Tumor volumes were assessed 2 weeks after the intradermal injection of tumor cells. A typical response is shown in Figure 2. Whereas tumors in WT mice averaged 540 mm ^3^, tumors in ACE 10/10 mice averaged only 90 mm ^3^. This difference was seen using different B16 sublines and in both inbred and outbred ACE 10/10 mice. The decrease in tumor growth was dependent on CD8 ^+^ T cells; in ACE 10/10 mice, depletion of CD8 ^+^ T cells, but not CD4 ^+^ T cells, led to rapid tumor growth. When B16 tumor cells constitutively expressing ovalbumin were used, tetramer analysis of T-cell receptors showed that ACE 10/10 mice have more circulating tumor-specific CD8 ^+^ T cells with specificity for the ovalbumin epitope SIINFEKL and the B16 TRP-2 epitope (SVYDFFVWL) than WT mice. Tumor resistance was dependent on ACE catalytic activity, as demonstrated by WT levels of tumor growth in ACE 10/10 mice treated with an ACE inhibitor ^13^. In contrast, treatment of the mice with an angiotensin II receptor antagonist had no effect on tumor growth.

An insight into the mode of tumor resistance in ACE 10/10 mice was provided by histological examination of the small tumors present in these mice. This revealed far larger numbers of inflammatory cells, including monocytes, macrophages, and some lymphocytes within the tumor blood vessels and the tumor itself than in tumors from WT mice. Furthermore, tumor resistance was transferable by bone marrow transplantation from ACE 10/10 into WT mice. This transplant experiment was important since WT mice chimeric for ACE 10/10 bone marrow have normal ACE expression in all tissues except bone marrow-derived cells. Thus, these data and several other lines of investigation indicated that it was the presence of ACE activity in bone marrow-derived cells, and not the lack of ACE expression in endothelium, that was important in the enhanced immune response of the ACE 10/10 mice.

The ACE 10/10 mice were also tested in a tumor metastasis model where the mice were injected intravenously with B16 tumor cells ^16^. After 14 days, melanotic modules in the lungs were quantitated. In this protocol, ACE 10/10 mice averaged only one third the number of visible lung metastases as in WT mice.

A detailed analysis of macrophage function in ACE 10/10 mice indicated that these cells have a pronounced pro-inflammatory “M1” phenotype, as compared with equivalent WT cells. Specifically, macrophages from ACE 10/10 mice produced more interleukin-12 (IL-12) p40, TNFα, and nitric oxide synthase II (inducible nitric oxide synthase, or iNOS) in response to tumor cells or LPS. On the other hand, the ACE 10/10 cells made less of the immunosuppressive cytokine IL-10 ^13, 17, 18^.

## Innate immunity in ACE 10/10 mice

Resistance to B16 melanoma is typically thought to be mediated by the adaptive immune response. In order to assess innate immunity, the resistance of the ACE 10/10 mice to infection with either *L. monocytogenes* or methicillin-resistant *S. aureus* (MRSA) was evaluated ^17^. Resistance to *L. monocytogenes* was assessed following intravenous injection of the strain EGD. Mice were sacrificed 3 or 5 days after infection, and bacterial counts were determined in the spleen and liver. This showed consistently fewer bacteria in ACE 10/10 mice as compared with WT. Pre-treatment of the ACE 10/10 mice for several days with the ACE inhibitor ramipril eliminated any significant differences between these mice and WT. In contrast, the angiotensin II receptor antagonist losartan had no significant effect.

Peritoneal macrophages were also tested *in vitro* for their ability to kill *L. monocytogenes*. In the absence of interferon-gamma (IFNγ) priming, there was no significant difference between ACE 10/10 and WT macrophages in the killing of *L. monocytogenes*. As ACE 10/10 macrophages express abundant surface ACE, these data demonstrate that ACE has no direct bactericidal effect. However, after IFNγ priming, ACE 10/10 macrophages were much more effective in killing bacteria than WT macrophages were ^17^.

ACE 10/10 mice were also challenged by intradermal infection with MRSA (USA300, strain SAF8300). After 4 days, bacterial counts in the skin lesions were determined. ACE 10/10 mice averaged 50-fold less bacteria within the MRSA lesions than WT mice. Again, this difference was eliminated by the ACE inhibitor lisinopril, and enhanced resistance to MRSA could be conferred by bone marrow transplant of ACE 10/10 bone marrow cells into WT mice ^17^.

## Immunological memory and antibody production

**Figure 3.**
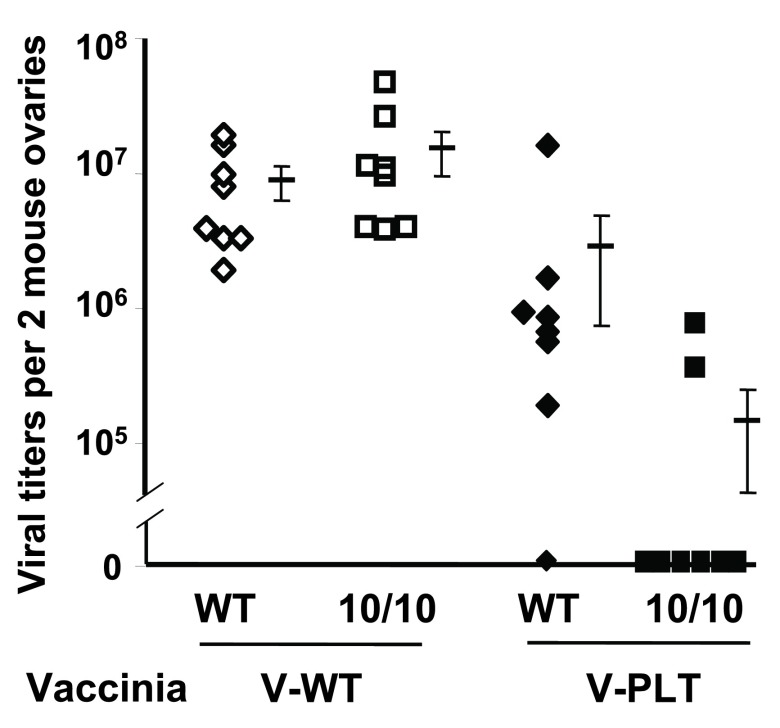
Polyoma virus recall. Wild-type (WT) and angiotensin-converting enzyme (ACE) 10/10 mice were infected with mouse polyoma virus. After 1 month, the mice were challenged with either WT vaccinia virus (V-WT) or a modified vaccinia virus which expressed a portion of the polyoma large T protein (V-PLT). Viral titers in the ovary were measured using a plaque assay. Whereas there is no difference in response to V-WT, the ACE 10/10 mice have a much better immune recall response to V-PLT.

To investigate immunological memory, female ACE 10/10 and WT mice were infected with polyoma virus. By 28 days, the mice clear serum polyoma and were re-challenged with either WT vaccinia virus (V-WT) or vaccinia virus modified to express the polyoma large T epitope LT359–368 (V-PLT) ^18^. Four days after V-WT infection, there was no difference in ovarian vaccinia viral titers as measured by a plaque assay ( Figure 3). However, when mice were challenged with V-PLT, there was a marked difference in viral titers. Thus, in a viral recall assay, these data indicate an increased immune response in the ACE 10/10 mice.

**Figure 4.**
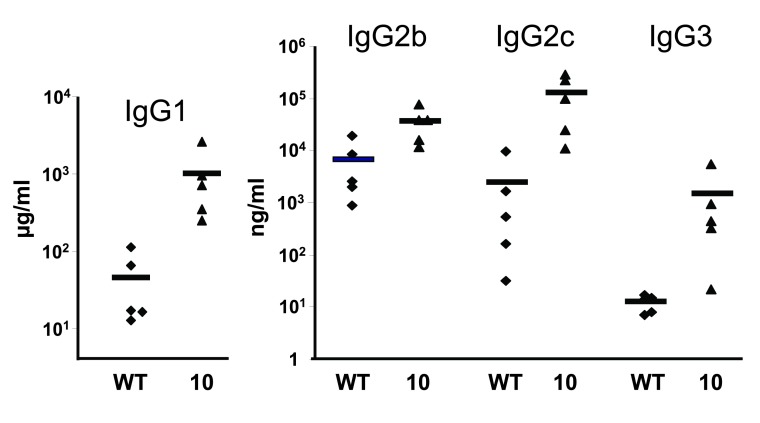
Anti-ovalbumin antibodies. Wild-type (WT) and angiotensin-converting enzyme (ACE) 10/10 mice were immunized subcutaneously with 100 μg of ovalbumin in complete Freund’s adjuvant. Ten days later, plasma was collected and titers of antibody subtypes specific to ovalbumin were determined by enzyme-linked immunosorbent assay. The major anti-ovalbumin antibody was IgG1, which showed a marked increase in the ACE 10/10 mice versus WT (990 versus 45 μg/mL). A similar pattern of increased antibody expression was seen for IgG2b, IgG2c, and IgG3 antibodies, though at lower levels of antibody expression. These data are for illustrative purposes only. The results are currently unpublished and have not been peer-reviewed but will be peer-reviewed and published elsewhere.

Finally, antibody production in ACE 10/10 mice was assessed after immunization with ovalbumin in complete Freund’s adjuvant ( Figure 4). Ten days after immunization, plasma was collected and titers of antibody subtypes specific to ovalbumin were determined by enzyme-linked immunosorbent assay. The major anti-ovalbumin antibody was IgG1, which showed a 22-fold increase in the ACE 10/10 mice versus WT. A similar pattern of antibody increase was seen for IgG2b, IgG2c, and IgG3 antibodies, though at substantially lower levels of antibody expression.

The data summarized here support the idea that ACE plays an important role in the immune response in addition to its role in regulating blood pressure. These data complement other studies implicating ACE in immunity to disease. These include a role of ACE and angiotensin II in the immune response to experimental auto-immune encephalomyelitis ^19– 22^, and rheumatoid and other types of arthritis ^23– 26^, in the production of the immuno-dominant epitope of the HIV protein gp160 ^27, 28^, and in generating the peptide repertoire diversity displayed by major histocompatibility complex (MHC) class I proteins ^29, 30^.

## Conclusions

ACE 10/10 mice are a model in which ACE is overexpressed in myelomonocytic cells, particularly monocytes and macrophages. It may be that this model recapitulates the expression of ACE by monocytic cells found in granuloma but that now supranormal expression levels generate a more pronounced immune phenotype than under natural conditions. We think that the most likely mechanistic explanation for the ACE 10/10 phenotype is that ACE overexpression fundamentally modifies the differentiation program of monocytes and macrophages. As indicated by the elimination of the phenotype after the administration of ACE inhibitors, the ACE 10/10 phenotype requires the catalytic activity of ACE and thus must be hypothesized to be a downstream effect of some ACE product(s). The precise peptides that have such a dramatic effect on the immune response are not known at present. The discovery that ACE overexpression enhances both innate and acquired monocytic function holds promise for an entirely new approach for improving the immune response to a variety of stimuli, including infections and tumors.

## Abbreviations

ACE, angiotensin-converting enzyme; IFNγ, interferon gamma; IL, interleukin; LPS, lipopolysaccharide; MRSA, methicillin-resistant *Staphylococcus aureus*; RAS, renin-angiotensin system; TNFα, tumor necrosis factor alpha; V-PLT, polyoma large T protein; V-WT, wild-type vaccinia virus; WT, wild-type.
